# Venous thrombosis related to duplicated inferior vena cava: A case report and systematic review

**DOI:** 10.1097/MD.0000000000041748

**Published:** 2025-02-28

**Authors:** Chun Chen, Di-Sheng Huang, Kuo-Song Chang, Yen-Chun Lai, Yu-Jang Su

**Affiliations:** a Division of Disaster Medicine, Emergency Department, Mackay Memorial Hospital, Taipei Taiwan; b Department of Emergency Medicine, HsinChu Mackay Memorial Hospital, HsinChu Taiwan; c Department of Nursing, MacKay Junior College of Medicine Nursing and Management, Taipei Taiwan; d Toxicology Division, Emergency Department, MacKay Memorial Hospital, Taipei, Taiwan; e Department of Anesthesiology, Taipei Medical University Hospital, Taipei City, Taiwan; f Department of Medicine, MacKay Medical College, New Taipei City, Taiwan; g Department of Nursing, Yuanpei University of Medical Technology, Hsinchu Taiwan.

**Keywords:** duplicated inferior vena cava, gender, venous thrombosis

## Abstract

**Rationale:**

Swollen legs are commonly observed in clinical practice, and deep vein thrombosis (DVT) is 1 of the causes. Duplicated IVC (DIVC) is a relatively rare anomaly that results in DVT.

**Patient concerns:**

A 52-year-old male patient presented to the emergency department because of right leg swelling, pain, and redness for 3 days. His right leg was swollen from the sole to the thigh, with mild tenderness and local heat, without pitting edema.

**Diagnoses:**

Laboratory tests showed mild elevated C-reactive protein (CRP) 3.82 mg/dL (reference value: 0–0.79 mg/dL), and notably high levels of D-dimer (25,700 ng/mL; reference value: 0–653). Lower limb computed tomography angiography showed duplication of the IVC (DIVC) and was highly suspicious for venous thrombosis involving the right popliteal vein, superficial femoral vein, common femoral vein, external iliac vein, internal iliac vein, common iliac vein, and inferior vena cava (IVC).

**Interventions:**

Enoxaparin (80 mg) was administered subcutaneously, twice daily. After admission, catheter-directed thrombolysis and thrombectomy via the right superficial femoral and popliteal veins were performed.

**Outcomes:**

The congestion in the right lower leg improved, and the patient was discharged with rivaroxaban 15 mg twice daily 3 days later.

**Lessons:**

A systematic review using the keywords “duplication IVC,” “thrombosis” and “case report” was performed on PubMed until May 2023. Males accounted for 55.2% (16 of 29) of the cohort. The mean ± standard deviation age of the patients was 48.9 ± 17.9 years old. Pulmonary embolism was documented in 27.6% (8 29) of the cases. Regarding treatments, we found that 21 patients (72.4%) used anticoagulants, 20 patients (69.0%) received IVC filter placement, and 7 cases (24.1%) were treated by catheter-directed thrombolysis. Women had a higher proportion of popliteal vein and calf vein thrombosis, with a statistically significant difference (25% vs 61.5%, *P* = .047 < .05), (12.5% vs 53.8%, *P* = .017 < .05). The incidence of DIVC is around 0.3% to 0.7% with male predominance. A DIVC is a risk factor for DVT, especially in young people.

## 1. Introduction

Duplicated inferior vena cava (DIVC) is a congenital anomaly of persistent left and right supra-cardinal veins, with an incidence of 0.7%.^[1]^ Deep vein thrombosis (DVT) and pulmonary embolism (PE) are possible clinical complications of this condition. Patients are often accidentally diagnosed with DIVC using computed tomography when examining the cause of DVT or PE. The initial management consisted of anticoagulation therapy to prevent thrombosis. Additional treatments such as catheter-directed thrombolysis, thrombectomy, or coil embolization of the duplicated segment may be beneficial.^[2–4]^ Thus, physicians should be aware of the possibility of duplication of the inferior vena cava (IVC) to prevent repeated thrombosis in the patient. In this study, we investigated the epidemiology, clinical complications of thrombosis, and presentation, treatment, and outcome of duplication of the IVC.

## 2. Case report

**Figure 1.**
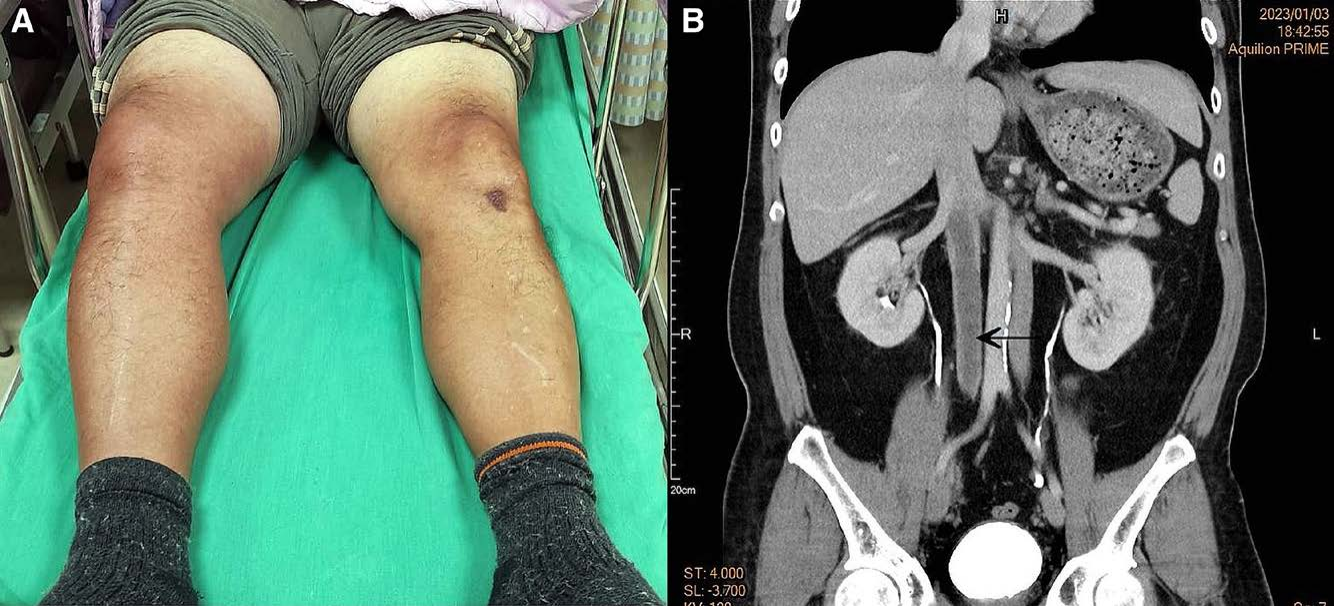
This 52-yr-old male suffered from the swollen right leg from sole to thigh, with mild tenderness and local heat without pitting edema.

A 52-year-old male patient presented to the emergency department because of right leg swelling, pain, and redness for 3 days. He denied fever, abdominal pain, dyspnea, chest pain, or palpitations. Physical examination revealed a body temperature of 36.3°C, pulse of 90 beats per minute, respiratory rate of 19 breaths per minute, and blood pressure of 133/92 mm Hg. His right leg was swollen from the sole to the thigh, with mild tenderness and local heat, without pitting edema (Fig. 1). Laboratory tests showed the white blood cell count was 11,400 with segmented neutrophils noted in 84.4%, mild elevated C-reactive protein (CRP) 3.82 mg/dL (reference value: 0–0.79 mg/dL), and notably high levels of D-dimer (25,700 ng/mL; reference value: 0–653). The coagulation function and platelet count were within normal limits. Lower limb computed tomography angiography showed duplication of the IVC (DIVC) and was highly suspicious for venous thrombosis involving the right popliteal vein, superficial femoral vein, common femoral vein, external iliac vein, internal iliac vein, common iliac vein, and inferior vena cava (IVC). Enoxaparin (80 mg) was administered subcutaneously, twice daily. After admission, catheter-directed thrombolysis and thrombectomy via the right superficial femoral and popliteal veins were performed. The congestion in the right lower leg improved, and the patient was discharged with rivaroxaban 15 mg twice daily 3 days later.

## 3. Systematic review

### 3.1. Materials and methods

#### 3.1.1. *Design and ethical approval*

This systemic review study was approved by the Institutional Review Boards of MacKay Memorial Hospital, Taipei, Taiwan (approval number: 23MMHIS209e). The research aimed to investigate the epidemiology, clinical complications of thrombosis, and the presentation, treatment, and outcomes related to duplication of the IVC. A systematic search using the keywords “duplication IVC,” “thrombosis,” and “case report” was conducted on PubMed up to May 2023, resulting in 30 potential articles. The study methodology followed the PRISMA 2020 guidelines.^[5]^

**Figure 2.**
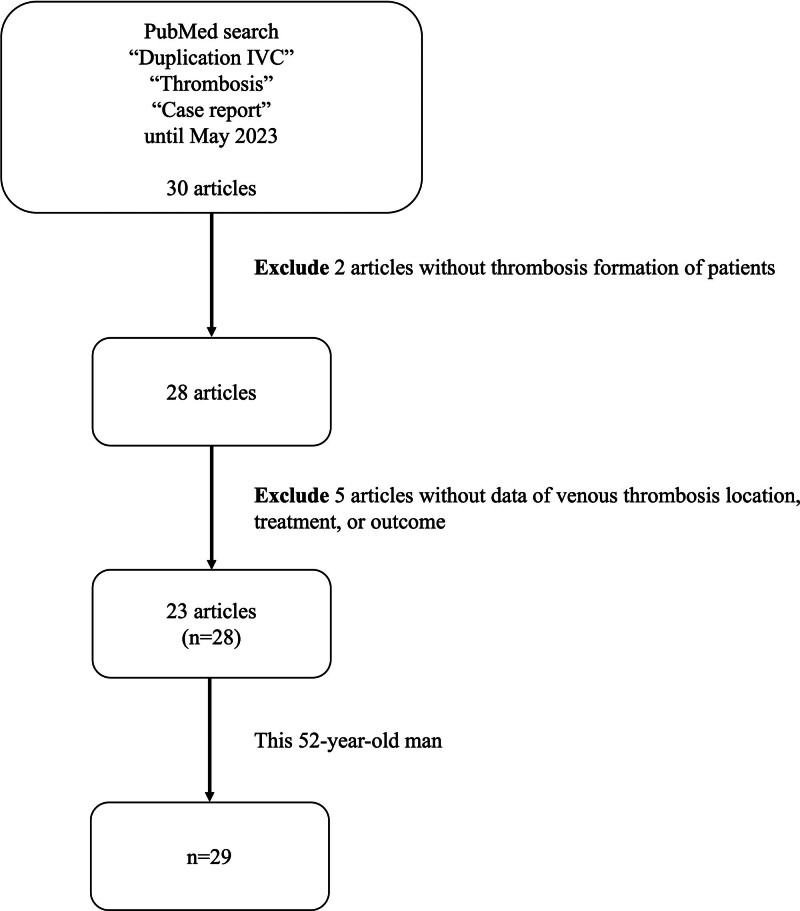
Flowchart of enrolled cases in this study.

Of the initial 30 articles, 2 case reports without thrombosis formation and 5 with incomplete data on thrombosis location, treatment, or mortality were excluded. Thus, 23 studies were selected for further analysis.^[4–27]^ Extracted data included: country, patient age, sex, thrombosis location, treatment (such as anticoagulant use, catheter-directed thrombolysis, and filter application), and mortality. A flowchart depicting the study enrollment process is provided in Figure 2.

### 3.2. *Statistical analyses*

The study also included a 52-year-old case previously mentioned, bringing the total number of cases to 29. Statistical analyses were performed using SPSS software (version 26.0). Continuous variables were analyzed using a 2-sample *t*-test, while categorical variables were evaluated with the chi-square test. A *P*-value of <.05 was considered statistically significant.

## 4. Results

After inspecting the retrieved studies, 23 case reports, comprising 28 patients, were identified as eligible. Of the retrieved studies, there were eleven cases were reported from the USA, 6 from China, 2 from Japan, India, and Canada, and 1 each from Iran, Oman, Osijek, Ireland, and Italy. The patient mentioned in this article was from Taiwan. Males accounted for 55.2% (16 of 29) of the cohort. The mean ± standard deviation age of the patients was 48.9 ± 17.9 years old in males and 55.3 ± 21.3 in females. PE was documented in 27.6% (8 29) of the cases. Right IVC thrombosis was documented in 3 cases, and the left IVC was documented in 1 case. There was 1 case of superior mesenteric vein thrombosis and 2 cases of renal vein thrombosis, all of which were male. Six patients had DVT without a specific location of the thrombus.

Regarding treatments, we found that 21 patients (72.4%) used anticoagulants, 20 patients (69.0%) received IVC filter placement, and 7 cases (24.1%) were treated by catheter-directed thrombolysis.

All patients were discharged alive and were followed up at the outpatient department. A comparison of presentations between non-elderly and elderly duplication IVC cases is presented in Table 1, it shows 79.3% were non-elderly, and 20.7% were elderly. Gender distribution was similar between the 2 groups, with no significant differences (*P* = .586). Clinical complications such as DVT, PE, and various vein thromboses were observed, but none of the differences reached statistical significance (*P* > .05). Treatment approaches, including filter insertion and anticoagulant use, were also similar between the groups, although non-elderly patients had a higher rate of catheter intervention (30.4%) compared to the elderly group (0%), with a *P*-value of .121. Overall, while there were some variations in clinical outcomes and treatments, the differences between non-elderly and elderly patients were not statistically significant.

**Table 1:** Comparison in presentations between non-elderly and elderly duplication IVC cases.

	Non-elderly (23, 79.3%)	Elderly ≥65 yr old (6, 20.7%)	*P*-value 2-tailed
Gender (M:F)	13:10	3:3	.586
DVT	(5, 21.7%)	(1, 16.7%)	.650
Pulmonary embolism	(6, 26.1%)	(2, 33.3%)	.278
Right IVC thrombosis	(2, 8.7%)	(1, 16.7%)	.426
SMV thrombosis	(0, 0%)	(1, 16.7%)	.180
Left IVC thrombosis	(1, 4.3%)	(0, 0%)	.180
Iliac vein thrombosis	(9, 39.1%)	(0, 0%)	.180
Renal vein thrombosis	(2, 8.7%)	(0, 0%)	.568
Femoral vein thrombosis	(14, 60.9%)	(1, 16.7%)	.351
Popliteal vein thrombosis	(11, 47.8%)	(1, 16.7%)	.719
Calf vein thrombosis	(7, 30.4%)	(2, 33.3%)	.663
Site of thrombosis (R: B: L)	9: 4: 10	4: 2: 0	.497
Filter insertion	(16, 69.6%)	(4, 66.7%)	.386
Intervention with catheter	(7, 30.4%)	(0, 0%)	.121
Anticoagulant usage	(17, 73.9%)	(4, 66.7%)	.702

DVT = deep vein thrombosis, IVC = inferior vena cava, NA = non-available, R:B:L = right:bilateral:left, SMV = superior mesenteric vein.

A comparison of the presentations of sex differences in DIVC is shown in Table 2, it shows 55.2% were male and 44.8% were female. The mean age was slightly higher in females (55.3 years) compared to males (48.9 years), though this difference was not statistically significant (*P* = .397). Notably, females exhibited significantly higher rates of popliteal and calf vein thrombosis (61.5% and 53.8%, respectively) compared to males (25% and 12.5%, with *P*-values of .047 and .017, respectively). There were no significant differences between genders in other thrombotic sites, treatments (filter insertion, catheter intervention, and anticoagulant use), or length of stay.

**Table 2:** Comparison in presentations between gender differences in duplication IVC cases.

	Male (16, 55.2%)	Female (13, 44.8%)	*P*-value 2-tailed
Age	48.9 ± 17.9	55.3 ± 21.3	.397
DVT	(5, 31.3%)	(1, 7.7%)	.119
Pulmonary embolism	(5, 31.3%)	(3, 23.1%)	.624
Right IVC thrombosis	(2, 12.5%)	(1, 7.7%)	.672
SMV thrombosis	(1, 6.3%)	(0, 0%)	.359
Left IVC thrombosis	(1, 6.3%)	(0, 0%)	.359
Iliac vein thrombosis	(6, 37.5%)	(3, 23.1%)	.404
Renal vein thrombosis	(2, 12.5%)	(0, 0%)	.186
Femoral vein thrombosis	(7, 43.8%)	(8, 61.5%)	.340
Popliteal vein thrombosis	(4, 25%)	(8, 61.5%)	.047
Calf vein thrombosis	(2, 12.5%)	(7, 53.8%)	.017
Site of thrombosis (R:B:L)	5: 4: 7	8: 2: 3	.389
Filter insertion	(10, 62.5%)	(10, 76.9%)	.404
Intervention with catheter	(4, 25%)	(3, 23.1%)	.904
Anticoagulant usage	(10, 62.5%)	(11, 84.6%)	.185
Length of stay, d	10.0 ± 8.8	12.0	.863

DVT = deep vein thrombosis, IVC = inferior vena cava, NA = non-available, R:B:L = right:bilateral:left, SMV = superior mesenteric vein.

## 5. Discussion

### 5.1. The etiologies, presentation, and management of DVT

Venous thromboembolism (VTE) occasionally complicates PE or deep venous thrombosis (DVT), affecting up to 5% of the population.^[28]^ PE develops when a thrombus dislodges from the vein walls and passes through the heart to the pulmonary arteries. DVT is the formation of a thrombus in the peripheral deep veins, most commonly the veins of the legs or pelvis.^[29]^ VTE can be categorized as either provoked or unprovoked. Unprovoked VTE is a non-environmental risk factor. Examples of non-environmental risk factors include hereditary thrombophilia, male sex, and older age.^[30]^ Provoked refers to a thrombotic event caused by an environmental or acquired risk factor. Active cancer, congestive heart failure, obesity, varicose veins, immobility, bed rest for more than 3 days, estrogen therapy, pregnancy, trauma, major surgery, and foreign objects or devices are provoked risk factors.^[31]^ The initial management for VTE was anticoagulation therapy. Prolonged use of anticoagulants or interventions should be considered when venous thromboembolism is due to unprovoked or persistent risk factors.^[32]^ The clinical manifestation of PE is sudden death in many patients. Post-thrombotic syndrome is a frequent complication of DVT and is characterized by chronic, persistent pain, heaviness, swelling, cramps, itching, tingling, or ulcers in the affected limb.^[33]^ A report from East Asia in 2023 described male sex, bilateral DVT, and elevated D-dimer levels as risk factors for IVC thrombosis occurrence.^[34]^

### 5.2. The incidence of DIVC is around 0.3% to 0.7% with male predominant

IVC duplication typically occurs below the renal veins. In most cases, the right IVC dominates with anastomoses from the left IVC at the level of the renal veins or occasionally drains directly into the left renal vein.^[35]^ Larger studies of patients undergoing CT scanning indicate that the prevalence of duplicate IVC is approximately 0.3% to 0.7%.^[1,36,37]^ In our study, 69% of cases received IVC filter treatment, and variants of congenital anomalies of the IVC and left renal vein were detected, such as azygos continuation of the IVC (0.1%), 3 duplicated IVCs (0.3%), and 10% left renal vein variants, including 3.7% retro-aortic renal veins and 6.3% circum-aortic venous rings. Evaluation with spiral CT and detailed knowledge of these anomalies are crucial for IVC filter placement.^[37]^ In our study, the male-to-female ratio was 1.2 without a significant statistical difference. In previous studies, analysis of 53 cases between 2000 and 2011 showed a slight male preponderance.^[38]^ Previous studies suggested that IVC anomalies should be considered in young patients with the spontaneous occurrence of blood clots^[17]^; however, previous studies did not report that this congenital variant was found in particularly young populations.

### 5.3. Old age and duplicated inferior vena cava are risk factors leading to deep vein thrombosis

Older age, male sex, obesity, history of diabetes, surgery history, duration of days in bed above 3 days, and a history of DVT are significant risk factors for venous reflux in patients with erythematous and swollen limbs.^[39–41]^ In another study enrolling patients with venous thromboembolism (VTE) after orthopedic foot and ankle surgery, female sex, increasing age, obesity, inpatient status, and nonelective surgery were all significantly associated with VTE.^[42]^ Congenital abnormality of the IVC is thought to be an anatomical risk factor for venous thromboembolism.^[21]^ Age ≥ 40 years and open surgery duration ≥ 30 min are all risk factors for thromboembolism.^[43]^

A United States study in 2013 reported that VTE occurred 5-fold after the age of 80 years; however, the incidence rate of VTE is approximately 1/1000.^[8]^ Some studies have suggested that the DIVC should be placed in the diagnostic list of patients under the age of 30 years with the spontaneous occurrence of blood clots.^[17]^

### 5.4. In duplicated inferior vena cava, female patient is more likely to happen to popliteal vein and calf vein thromboembolism than the males

A United States study in 2008 described DVT incidence to be similar in men and women.^[17]^ In 2019, a United States report described that female sex, increasing age, obesity, inpatient status, and nonelective surgery were all significantly associated with venous thromboembolism (VTE) after ankle and foot surgery.^[42]^ No research has depicted the difference in the location of VTE by sex. However, in our study, the female gender was more likely to occur in the popliteal vein (2.5-fold) and calf vein (4.3-fold) VTE than in males. This conclusion is the first in the world to describe this gender difference. A Saudi Arabian report in 2023 also disclosed that DVTs are more prevalent in females than males (60% vs 40%) and most commonly affect patients older than 40.^[44]^

### 5.5. Duplicated inferior vena cava is a risk factor leading to deep vein thrombosis, especially in young people

Au US report in 2011 describing anatomic variations of the IVC are found in 3% to 5% of the population, and IVC thrombosis ranges from 60% to 80% among patients with congenital IVC anomalies.^[6,10]^ IVC anomalies have become a considerable risk factor for DVT in the lower extremities, especially in young patients. In another study in 2017 from Serbia, 11.1% of IVC anomalies developed DVT in the left calf vein.^[45]^

## 6. Limitation

The DIVC is a seldom seen vascular anomaly with an incidence of 0.3% to 0.7% in the population, so that consecutive data can be gathered via multi-center or national registry data and prevent data collection bias. Second, data retrieved from PubMed articles and not every case reporting sufficient parameters or items to be compared is another limitation of this research.

## 7. Conclusion

The risk factors for venous thromboembolism are hereditary thrombophilia, male sex, older age, heart failure, obesity, varicose veins, immobility, bed rest for more than 3 days, estrogen therapy, pregnancy, trauma, major surgery, and foreign objects or devices. The incidence of DIVC is around 0.3% to 0.7% with male predominance, but female patients are more likely to develop popliteal vein and calf vein thromboembolism than male patients, and DIVC itself is a risk factor for VTE.

A DIVC is a risk factor for DVT, especially in young people.

## Acknowledgments

We thank the attending physician Pang-Yen Chen for raising the attention of this case to be educatable.

## Author contributions

**Conceptualization:** Kuo-Song Chang, Yen-Chun Lai.

**Data curation:** Chun Chen, Di-Sheng Huang.

**Formal analysis:** Yu-Jang Su.

**Investigation:** Chun Chen, Yen-Chun Lai, Yu-Jang Su.

**Methodology:** Chun Chen, Yu-Jang Su.

**Project administration:** Yu-Jang Su.

**Resources:** Yu-Jang Su.

**Software:** Kuo-Song Chang, Yu-Jang Su.

**Supervision:** Yu-Jang Su.

**Validation:** Yu-Jang Su.

**Visualization:** Yen-Chun Lai, Yu-Jang Su.

**Writing – original draft:** Chun Chen, Di-Sheng Huang, Kuo-Song Chang, Yu-Jang Su.

**Writing – review & editing:** Yen-Chun Lai, Yu-Jang Su.
